# Hyphema is a risk factor for failure of trabeculectomy in neovascular glaucoma: a retrospective analysis

**DOI:** 10.1186/1471-2415-14-55

**Published:** 2014-04-26

**Authors:** Shunji Nakatake, Shigeo Yoshida, Shintaro Nakao, Ryoichi Arita, Miho Yasuda, Takeshi Kita, Hiroshi Enaida, Yuji Ohshima, Tatsuro Ishibashi

**Affiliations:** 1 Department of Ophthalmology, Graduate School of Medical Sciences, Kyushu University, 3-1-1 Maidashi, Higashi-Ku, Fukuoka 812-8582, Japan

**Keywords:** Neovascular glaucoma, Trabeculectomy, Hyphema, Mitomycin C, Intravitreal bevacizumab

## Abstract

**Background:**

Several retinal ischemic diseases can cause neovascular glaucoma (NVG). Trabeculectomy with mitomycin C (MMC) is a relatively better treatment modality in the management of eyes with NVG than other glaucoma surgeries. The aim of this study was to investigate the factors that may influence the outcome of trabeculectomy with MMC for NVG.

**Methods:**

Forty-nine NVG eyes from 43 patients (26 males and 17 females) underwent primary trabeculectomy with MMC. The mean follow-up period was 16.8 ± 8.1 months (range, 6 to 34 months). Twenty-one eyes of 21 patients received intravitreal bevacizumab (IVB) 3.6 ± 1.8 days before trabeculectomy with MMC. A Kaplan-Meier survival-curve analysis was used to summarize the cumulative probability of success. We examined the relationship between the surgical outcome and the following surgical factors: gender, age, history of panretinal photocoagulation, history of cataract surgery, history of vitrectomy, preoperative IVB, NVG in the fellow eye, and postoperative complications (hyphema, choroidal detachment, and formation of fibrin) by multivariate analysis.

**Results:**

The survival rate was 83.7% after 6 months, 70.9% after 12 months, and 60.8% after 24 months. The Kaplan-Meier survival curves showed no significant difference in the survival rate between the eyes with preoperative IVB (n = 21) and the eyes without preoperative IVB (n = 28) (p = 0.14). The multiple logistic regression analysis showed that postoperative hyphema (odds ratio, 6.54; 95% confidence interval, 1.41 to 35.97) was significantly associated with the surgical outcome (p = 0.02).

**Conclusions:**

Postoperative hyphema was significantly correlated with the outcome of trabeculectomy for NVG. There was no significant association between preoperative IVB and postoperative hyphema or the results of trabeculectomy.

## Background

Several retinal ischemic diseases can cause neovascular glaucoma (NVG): proliferative diabetic retinopathy (PDR), central retinal vein occlusion (CRVO), branch retinal vein occlusion (BRVO), central retinal artery occlusion (CRAO), and ocular ischemic syndrome (OIS) [1]. Patients with NVG generally present with elevated intraocular pressure (IOP), hyphema, and vitreous hemorrhage. If the elevation in IOP is severe the patient may experience severe pain, and the elevated IOP often results in disastrous visual loss.

NVG is a severe form of glaucoma characterized by neovascularization and the proliferation of fibrovascular tissue in the anterior chamber angle. In the early open-angle glaucoma stage, anti-glaucoma drugs or panretinal photocoagulation (PRP) may be effective. However, as the disease progresses, the proliferative fibrovascular membrane causes angle closure. This stage is not reversible by PRP and is often refractory to anti-glaucoma drugs. Trabeculectomy with mitomycin C (MMC) is a good treatment modality in the management of eyes with NVG [2-4], but its success rate is still poor [5].

The pathogenesis of NVG is related to the production of vascular endothelial growth factor (VEGF) by the underlying ischemic retina, which stimulates neovascularization in the anterior chamber angle [1,6]. Bevacizumab is a human monoclonal antibody that binds VEGF and blocks its action. Intravitreal bevacizumab (IVB) was reported to decrease the concentration of VEGF in the aqueous humor [7], the neovascularization of the anterior chamber, and the IOP to acceptable levels in NVG patients [8-12]. Fluorescein angiography and histochemical investigations demonstrated that there was less vascular permeability and inflammatory reaction in trabecular tissue with IVB than without IVB [13,14]. Another histopathological investigation indicated that IVB may induce changes in immature, newly formed vessels, leading to endothelial apoptosis with vascular regression and inducing the normalization of premature vessels in PDR or NVG eyes [15]. Therefore, IVB before trabeculectomy surgery is expected to have potential as a surgical adjuvant to reduce operative bleeding complications.

According to previous reports, patient factors such as previous PRP [16] and history of preoperative IVB [16,17] were indicated as good prognostic factors for the surgical outcome of trabeculectomy for NVG, whereas a history of vitrectomy [3,18], and younger age [18] were indicated as adverse prognostic factors. However, it is not yet known which factors are definite prognostic factors [3,18]. In addition, to the best of our knowledge there has been no report about the effect of early postoperative hyphema on the success rate of trabeculectomy with NVG. In the present study, we investigated the surgical prognostic factors of trabeculectomy.

## Methods

### Patients

We retrospectively reviewed the medical records of 49 NVG eyes from 43 patients (26 males and 17 females) with the presence of neovascularization in the anterior chamber angle and uncontrolled IOP. All patients underwent primary trabeculectomy with MMC at Kyushu University Hospital, Japan, between January 2008 and July 2010 and could be followed up for more than 6 months. This study was approved by the Ethics Committee of Kyushu University (Ethics Approval Number: 19002). No patients underwent any other glaucoma surgery before the primary trabeculectomy. The etiology of NVG was PDR (43 eyes), CRVO (2 eyes), and CRAO, BRVO, OIS and uveitis (1 eye each). PRP was performed as much as possible except for patients who could not undergo PRP because of vitreous hemorrhage or corneal edema. Excluding those with a history of ischemic cardiac disease or brain infarction and in poor general status, 21 eyes of 21 patients received IVB (1.25 mg/0.05 mL) at 1 to 7 (3.6 ± 1.8) days before trabeculectomy with MMC after 2009 March.

### Surgical technique

All 43 patients underwent trabeculectomy with MMC. A fornix-based conjunctival flap was made. After hemostasis of the episcleral blood vessels with wet-fluid cautery, a 3 × 3-mm, half-thickness triangle scleral flap was made. Surgical sponges soaked in MMC (0.4 mg/mL) were placed under the conjunctival flap for 3 min, followed by irrigation with 300 mL of physiologic saline. A deep trabecular block was removed and a peripheral iridectomy was performed. The scleral flap was sutured with 10-0 nylon sutures. All patients received topical treatment including 0.5% levofloxacin and 0.1% betamethasone for 1 to 3 months after the operation, and the patients whose IOP was elevated received anti-glaucoma drugs. Postoperatively, argon laser suture lysis was performed, depending on the level of IOP and the condition of the conjunctival bleb formation.

### Data analysis

The criteria for successful surgery were: IOP < 20 mmHg with or without anti-glaucoma drugs, visual acuity more than light perception, and no additional glaucoma surgery (shunt insertion or cyclophotocoagulation). The following patient data were collected: age, gender, preoperative and postoperative (1 week, 1, 2, 3, and 6 months after the surgery, and last visit) IOP, lens status, previous surgical ocular history (except for glaucoma surgery), preoperative and postoperative visual acuity, the number of preoperative and postoperative anti-glaucoma drugs the patient had been taking, and postoperative complications.

Hyphema, one of the potential postoperative complications of trabeculectomy, was defined as hemorrhage in the anterior chamber to the extent of making niveau, even if only a little. Eyes that showed only suspended red blood cells in the anterior chamber, without making niveau, were not defined as having hyphema. The patients’ visual acuity was measured by assessing their decimal visual acuity, and it was calculated after conversion to the logarithm of the minimum of resolution (logMAR).

A Kaplan-Meier survival curve was used to estimate the probability of the success rate. We examined the relationships between the surgical risk factors and the surgical outcome. We considered the following ten possible risk factors for surgical outcome: gender, age, history of previous PRP, history of cataract surgery, history of vitrectomy, concurrent vitrectomy, preoperative IVB, NVG in the fellow eye, postoperative hyphema, postoperative choroidal detachment, and postoperative formation of fibrin. All variables were treated as categorical variables. Each categorical variable was coded as either 1 or 0 depending on the presence or absence of the factor, respectively. Frequencies were compared by chi-square test.

We estimated the multivariate odds ratio (OR) and 95% confidence interval (CI) of the following three potential risk factors by using a logistic regression analysis: history of vitrectomy [3,18], preoperative IVB [16,17], both of which have been reported to affect the surgical outcome, and postoperative hyphema. The JMP version 9 statistical package program (Cary, NC) was used to perform the statistical analyses. A two-sided p-value of less than 0.05 was considered significant.

## Results

Table 1 shows the baseline characteristics of all 49 eyes. The mean age of the patients was 55.6 ± 11.5 years with a range from 30 to 79 years. Twelve patients (14 eyes) were less than 50 years old, and 31 patients (35 eyes) were ≥ 50 years old. The mean follow-up period was 16.8 ± 8.1 months with a range from 6 to 34 months. All eyes were followed up for 6 months or more; 34 eyes were followed for 12 months or more, and 12 eyes were followed for 24 months or more. The mean preoperative IOP was 30.4 ± 13.1 mmHg, and the mean last-visit IOP was 16.0 ± 9.9 mmHg. Forty-two eyes received PRP. The other 7 eyes could not undergo sufficient retinal photocoagulation because of vitreous hemorrhage or corneal edema. Twenty-one eyes underwent preoperative IVB at 3.6 ± 1.8 (range 1–7) days before trabeculectomy.

**Table 1:** Baseline characteristics of 43 patients (49 eyes) who underwent trabeculectomy with MMC for NVG

**Characteristic**	**Value**
Gender	
Male	26 (60.5%)
Female	17 (39.5%)
Age (years)	
Mean ± SD	55.6 ± 11.5
Range	30–79
Follow-up (months)	
Mean ± SD	16.8 ± 8.1
Range	6–34
Preoperative IOP (mmHg)	
Mean ± SD	30.4 ± 13.1
Range	7–60
Prior surgery	
Cataract	39 (79.6%)
Pseudophakia	37 (75.5%)
Aphakia	2 (4.1%)
Vitrectomy	30 (61.2%)
20-gauge system	28 (57.1%)
23-gauge system	2 (4.1%)
Concurrent vitrectomy	
Yes	25 (51.0%)
No	24 (49.0%)
NVG in fellow eye	
Yes	13 (26.6%)
No	36 (73.4%)
Anti-glaucoma drugs	
Preoperative (mean ± SD)	2.4 ± 0.9
Postoperative (mean ± SD)	1.4 ± 1.3
Previous PRP	
Yes	42 (85.7%)
No	7 (14.3%)
Preoperative IVB	
Yes	21 (42.9%)
No	28 (57.1%)

MMC: mitomycin C.

Table 2 shows the postoperative complications of trabeculectomy with MMC in patients with NVG. Eleven eyes developed hyphema: 9 eyes at postoperative 1 day, 1 eye at postoperative 2 days, and 1 eye at postoperative 4 days. Four eyes had developed a formation of fibrin in the anterior chamber, and 5 eyes had developed choroidal detachment as postoperative surgical complications. Of all 49 eyes, 35 eyes were successes, and 14 eyes were failures.

**Table 2:** Postoperative complications

**Disease**	**Eyes (%)**	**Eyes**	
		**IVB**	**not IVB**
Hyphema	11 (22.4%)	6	5
Formation of fibrin	4 (8.2%)	0	4
Choroidal detachment	5 (10.2%)	1	4

**Figure 1:**
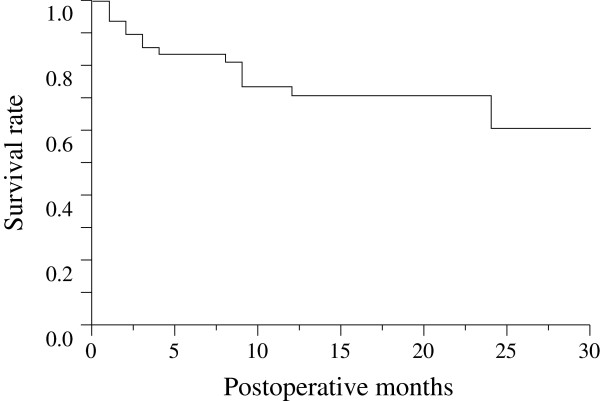
**Kaplan-Meier survival curve of all eyes.** The survival rate was 83.7% after 6 months, 70.9% after 12 months, and 60.8% after 24 months.

**Figure 2:**
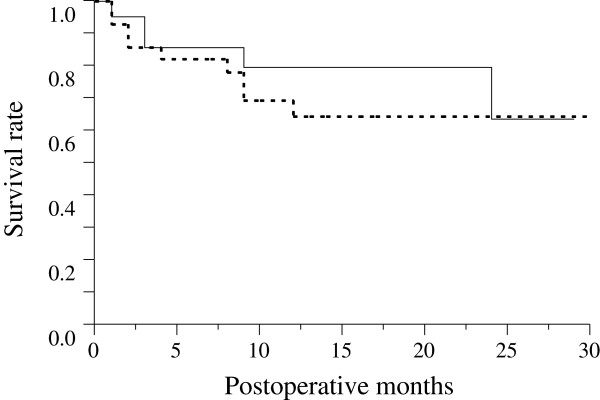
**Kaplan-Meier survival curve for IVB group vs. not-IVB group.** Kaplan-Meier survival curve of the surgical outcomes of trabeculectomies with MMC in eyes with IVB (n = 21, continuous line) and eyes without IVB (n = 28, dotted line). There was no significant difference between the two groups (p = 0.41).

Figure 1 is the Kaplan-Meier survival curve of all eyes. All 49 eyes were evaluated after 6 months, 34 eyes were evaluated after 12 months, and 12 eyes were evaluated after 24 months. The survival rate was 83.7% after 6 months, 70.9% after 12 months, and 60.8% after 24 months. Figure 2 is the Kaplan-Meier survival curve for the two groups (IVB group vs. not-IVB group). Until 23 months after the trabeculectomy, the survival rate of the IVB group was higher than that of the not-IVB group, but there was no significant difference between the two groups (p = 0.41).

**Figure 3:**
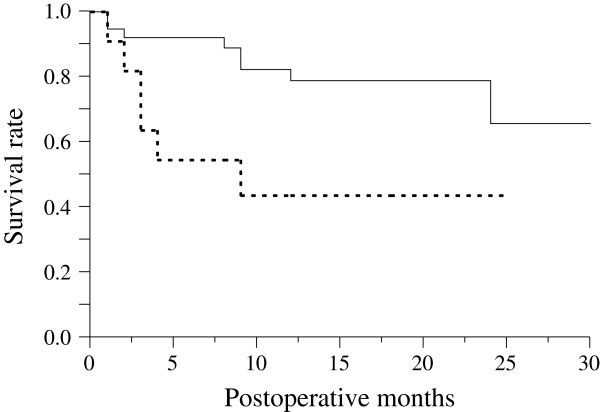
**Kaplan-Meier survival curve for the groups with hyphema vs. without hyphema.** Kaplan-Meier survival curve of the surgical outcomes of trabeculectomies with MMC in eyes with hyphema (n = 11, continuous line) and eyes without hyphema (n = 38, dotted line). There was a significant difference between the two groups (p < 0.01).

Figure 3 is the Kaplan-Meier survival curve of the surgical outcomes of the trabeculectomies with MMC in eyes with and without hyphema. The survival rate of the group without hyphema was significantly higher than the group with hyphema (p < 0.01). Table 3 shows the frequencies of the surgical risk factors that potentially influence the results of trabeculectomy for NVG. The frequency of postoperative hyphema was significantly higher in the failure group than in the success group. Table 4 shows the results of the multivariate analysis for the factors that influenced the surgical outcome. In the multivariate analysis, postoperative hyphema was significantly associated with the surgical outcome (p < 0.02).

**Table 3:** Frequencies of surgical risk factors that influence the result of trabeculectomy with MMC for NVG

**Variable:**	**Success group (35 eyes) Number of eyes (Percentage of eyes)**	**Failure group (14 eyes) Number of eyes (Percentage of eyes)**	**p-value (Chi-square test)**
Gender (Male)	20	9	0.65
	(57.1%)	(64.3%)	
Age (50<)	9	5	0.49
	(25.7%)	(35.7%)	
History of previous PRP	30	12	1.00
	(85.7%)	(85.7%)	
History of cataract surgery	29	10	0.37
	(82.9%)	(71.4%)	
History of vitrectomy	23	7	0.31
	(65.7%)	(50.0%)	
Concurrent vitrectomy	18	7	0.93
	(51.4%)	(50.0%)	
Preoperative IVB	16	5	0.52
	(45.7%)	(35.7%)	
NVG in the fellow eye	9	4	0.84
	(25.7%)	(28.6%)	
Postoperative hyphema	5	6	0.03
	(14.3%)	(42.9%)	
Postoperative choroidal detachment	4	1	0.65
	(11.4%)	(7.1%)	
Postoperative formation of fibrin	2	2	0.32
	(5.7%)	(14.3%)	

**Table 4:** Multivariate analysis of the factors that influence the result of trabeculectomy with MMC for NVG

**Variable**	**Odds ratio**	**95% CI**	**p-value**
History of vitrectomy	0.26	0.05–1.21	0.09
Preoperative IVB	0.28	0.05–1.30	0.10
Postoperative hyphema	6.54	1.41–35.97	0.02

CI, confidence interval.

## Discussion

Even with some therapies, the IOP is often increased to an uncontrolled level, sometimes leading to blindness. According to a systematic review and meta-analysis of comparative studies of two or more surgical techniques (one of which had to be trabeculectomy), including patients with open-angle glaucoma, trabeculectomy still offers the possibility of obtaining excellent IOP control at the long-term follow-up in patients with open-angle glaucoma [19], whereas the success rate of trabeculectomy for NVG is still poor [5]. In the present study, the survival rate of trabeculectomy for NVG was 83.7% after 6 months, 70.9% after 12 months, and 60.8% after 24 months.

Several previous studies investigated the success rate of trabeculectomy with MMC for NVG. One study reported that the cumulative probability of the success of trabeculectomy was 67.0% after 1 year and 61.8% after 2 to 3 years [3]. According to another report, the probability of success at 120, 240 and 360 days after combined preoperative IVB and trabeculectomy was 87.5%, 79.2%, and 65.2%, respectively, but the corresponding values after only trabeculectomy without IVB were 75.0%, 71.9%, and 65.3%, respectively [20]. Our surgical outcomes in the present study are similar to these reports.

Although there was a trend for less failure in the IVB group until postoperative 23 months, preoperative IVB finally did not significantly improve the outcome of trabeculectomy with MMC for NVG in our study. Moreover, it did not decrease the risk of postoperative hyphema. Sugimoto et al. [21], using resected irises from NVG patients during trabeculectomy, reported that IVB reduced the neovascularization on the iris surface but could not completely eliminate neovascularization in the iris stroma. They also reported that IVB did not prevent postoperative complications at 1 day after trabeculectomy, including hyphema [21]. Takihara et al., performing IVB 1 to 5 days before the trabeculectomy for NVG patients, reported that preoperative IVB did not significantly improve the surgical outcomes, in common with our study [20].

In contrast, Saito et al. reported that preoperative IVB decreased postoperative hyphema and increased the surgical success rate [17]. The discrepancy between these studies may be due to the different intervals between the IVB and the trabeculectomy: in the Sugimoto study and Takihara study, they performed the IVB 6 to 8 days and 1 to 5 days before the trabeculectomy, respectively, whereas in the Saito study, the IVB was performed 10 ± 11 days before the trabeculectomy. In our study, the IVB was performed 3.6 ± 1.8 days before the trabeculectomy, and the results are consistent with the Sugimoto study and Takihara study. Considering these data, using a sufficiently long interval between the IVB and trabeculectomy may be more effective by calming down the activity of neovascularization in the anterior segment.

Another study demonstrated that NVG eyes with a history of vitrectomy had poor surgical outcomes after trabeculectomy [22]. Vitrectomy exacerbates retinal ischemia and increases the concentration of inflammatory cytokines and/or VEGF in the anterior chamber [3]. Our present analysis did not find that previous vitrectomy is a prognostic factor. Thirty eyes had already undergone a vitrectomy before trabeculectomy, and 25 eyes underwent a vitrectomy concurrently to receive more photocoagulation to the extreme periphery of the retina intraoperatively. Additional photocoagulation may improve retinal ischemia; here it may have led to the satisfactory surgical outcomes. Moreover, some reports indicated that younger NVG patients have a poor success rate following trabeculectomy [18], but in the present study we found no significant difference in the surgical outcome between patients under 50 years old (14 eyes) and those ≥ 50 years old (35 eyes). This may be because there were many more patients over 50 years old in our study.

Trabeculectomy in NVG patients usually results in frequent postoperative complications and poor surgical outcomes. Hyphema has been described as the most frequent postoperative complication of trabeculectomy in NVG patients [16]. However, there is no report that postoperative hyphema significantly influences the surgical outcome of trabeculectomy with NVG. In the present study, hyphema was the most common postoperative complication, and we also found that postoperative hyphema was a significant prognostic factor in trabeculectomy for NVG. Moreover, patients with PDR were found in another study to have significantly higher serum levels of cytokines such as interleukin (IL)-6, tumor necrosis factor-alpha (TNF-α and VEGF compared to non-PDR patients [23]. The serum levels of TNF-α, IL-6, and C-reactive protein (CRP) were also higher in subjects with arteriosclerotic peripheral vascular disease compared to healthy controls [24].

TNF and IL-1 were reported to be capable of stimulating the proliferation of Tenon’s capsule fibroblasts [25]. Moreover, Cvenkel et al. indicated that lower levels of TNF-α and IL-6 in the aqueous humor were associated with better surgical outcomes in patients undergoing trabeculectomy [26]. Tripathi et al. reported the results of an intracameral injection of tissue plasminogen activator in the anterior chamber for eyes with fibrin clots and elevated IOP after glaucoma filtering procedures [27]; soon after the injection, the fibrin clots dissolved completely and the elevated IOP values decreased to the normal level. Tripathi et al. suggested that fibrin clots after filtering surgery provide a scaffold for the formation of scar tissue in the anterior chamber and in the fistulization tract.

In view of these reports, when hyphema occurs after trabeculectomy, increased concentrations of some cytokines may lead to a failure of conjunctival bleb formation. Additionally, blood flow into the trabecular meshwork may lead to a stronger wound-healing response or clog the flow of aqueous humor, but further study is required to test this notion.

There are a few reports about reducing the incidence of hyphema following trabeculectomy. Wilson et al. reported that during trabeculectomy, filling the anterior chamber with sodium hyaluronate reduced the incidence of postoperative hyphema for glaucoma patients with primary open-angle glaucoma, chronic angle closure glaucoma, congenital glaucoma, or NVG [28]; they suggested that the reduced incidence was due to a microvascular clotting effect or the tamponade of higher intraocular pressure. To avoid unfavorable hyphema, Elgin et al. used direct cauterization of the iris before iridectomy, and they reported that this protocol effectively reduced the rate of intraoperative bleeding and postoperative hyphema in trabeculectomy for NVG [29]. The direct cauterization of the iris seeking to reduce postoperative bleeding may be a beneficial new procedure for better prognosis in NVG patients.

In addition, as a new glaucoma surgery, the Ex-PRESS drainage device was introduced in 2011 as an alternative to trabeculectomy [30]. During this operation, iridectomy is not required, and thus hyphema is less likely compared to the use of trabeculectomy. The results of our present retrospective study indicate that postoperative hyphema could be a surgical risk factor for failure of trabeculectomy in NVG, but in light of the study’s retrospective nature, further prospective randomized investigations are required. Filling the anterior chamber with ophthalmic viscosurgical devices intraoperatively, the direct cauterization of the iris, or the use of the Ex-PRESS drainage device may provide better prognoses in NVG patients.

## Conclusions

In the present study, we found that postoperative hyphema was a significant prognostic factor in trabeculectomy for NVG. There was no clear association between preoperative IVB and postoperative hyphema or the result of trabeculectomy.

## Abbreviations

NVG: Neovascular glaucoma; MMC: Mitomycin C; IVB: Intravitreal bevacizumab; PDR: Proliferative diabetic retinopathy; CRVO: Central retinal vein occlusion; BRVO: Branch retinal vein occlusion; CRAO: Central retinal artery occlusion; OIS: Ocular ischemic syndrome; IOP: Intraocular pressure; PRP: Panretinal photocoagulation; VEGF: Vascular endothelial growth factor; OR: Odds ratio; CI: Confidence interval.

## Competing interests

The authors declare that they have no competing interests.

## Authors’ contributions

SN participated in the design of the study, performed the statistical analysis and drafted the manuscript. SY is the main researcher and the corresponding author. SN and RA recorded the data of the patients. MY participated in collecting the data and in the statistical analyses. TK, HE and YO drafted and revised the manuscript. TI participated in the design and final approval of manuscript. All authors read and approved the final manuscript.

## Pre-publication history

The pre-publication history for this paper can be accessed here:

http://www.biomedcentral.com/1471-2415/14/55/prepub
